# Analysis of factors influencing weight regain after bariatric-metabolic surgery in obesity hypoventilation syndrome patients based on gastrointestinal peptide hormones

**DOI:** 10.5937/jomb0-57277

**Published:** 2025-11-05

**Authors:** Jingjing Zhang, Shadike Apaer, Shuo Zhang, Guanyou Liang, Tao Li, Xinling Cao

**Affiliations:** 1 State Key Laboratory of Pathogenesis, Prevention and Treatment of High Incidence Diseases in Central Asia, Department of Nephrology, The Fist Affiliated Hospital of Xinjiang Medical University, Urumqi, Xinjiang, 830054, China; 2 State Key Laboratory of Pathogenesis, Prevention and Treatment of High Incidence Diseases in Central Asia, Department of Liver Transplantion & Laparoscopic Surgery, The First Affiliated Hospital of Xinjiang Medical University, Urumqi, Xinjiang, 830054, China

**Keywords:** gastrointestinal peptide hormones, weight regain, laparoscopic sleeve gastrectomy, obesity hypoventilation syndrome, glucagon-like peptide-1, gastrointestinalni peptidni hormoni, vracanje težine, laparoskopska gastrektomija rukava, sindrom hipoventilacije gojaznosti, peptid sličan glukagonu-1

## Abstract

**Background:**

Obesity is becoming increasingly prevalent in modern society, leading to a rise in the incidence of obesity hypoventilation syndrome (OHS). This study analyzes the factors influencing weight regain in OHS patients following laparoscopic sleeve gastrectomy (LSG), based on gastrointestinal peptide hormones.

**Methods:**

A total of 134 OHS patients who underwent LSG at our hospital between January 2023 and January 2024 were enrolled. The alterations in gastrointestinal peptide hormones, including insulin (INS), leptin (Lep), glucagonlike peptide-1 (GLP-1), and ghrelin (GHR), before and after surgery were measured. Subsequently, a 6-month followup was conducted. Patients with weight regain were identified, and the predictive value of gastrointestinal peptide hormones for weight regain was analyzed. Logistic regression was then employed to analyze the related factors affecting weight regain.

**Results:**

Following the surgical procedure, a significant increase was observed in the levels of INS, Lep, and GLP accompanied by a notable decrease in GHR levels among the patients (P &lt; 0.05). During the follow-up period, 32 patients experienced weight regain. The receiver operating characteristic (ROC) curve analysis demonstrated that gastrointestinal peptide hormones exhibited outstanding predictive capabilities for postoperative weight regain. Moreover, through statistical analysis, it was determined that unfavorable dietary habits, lack of regular exercise, trace element deficiencies, and negative emotional states were independent risk factors influencing weight regain following LSG (P &lt; 0.05).

**Conclusions:**

There is a close correlation between gastrointestinal peptide hormones and post-LSG weight alterations in patients with OHS.

## Abstract (sr)

**Uvod:**

Gojaznost postaje sve prisutnija u modernom drustvu, sto dovodi do porasta incidence hipoventilacionog sindroma gojaznosti (OHS). Ova studija analizira faktore koji utiču na ponovno dobijanje telesne težine kod pacijenata sa OHS nakon laparoskopske sleeve gastrektomije (LSG), na osnovu gastrointestinalnih peptidnih hormona.

**Metode:**

Ukupno je upisano 134 pacijenata sa OHS koji su podvrgnuti LSG u nasoj bolnici u periodu od januara 2023. do januara 2024. godine. Merene su promene u gastrointestinalnim peptidnim hormonima, uključujuci insulin (INS), leptin (Lep), peptid sličan glukagonu-1 (GLP-1) i grelin (GHR), pre i posle operacije. Nakon toga, sprovede-no je 6-mesečno pracenje. Identifikovani su pacijenti sa povratkom telesne težine i analizirana je prediktivna vred-nost gastrointestinalnih peptidnih hormona za vracanje telesne težine. Zatim je koriscena logistička regresija da se analizira-ju povezani faktori koji utiču na ponovno dobijanje težine.

**Rezultati:**

Nakon hirurske procedure primeceno je zna-čajno povecanje nivoa INS, Lep i GLP praceno značajnim smanjenjem nivoa GHR kod pacijenata (P &lt; 0,05). Tokom perioda pracenja, 32 pacijenta su doživela povratak težine. Analiza krive radne karakteristike prijemnika (ROC) pokaza-la je da gastrointestinalni peptidni hormoni pokazuju izvan-redne prediktivne sposobnosti za postoperativni povratak težine. Staviše, statističkom analizom utvrđeno je da su nepovoljne navike u ishrani, nedostatak redovnog vežba-nja, nedostaci elemenata u tragovima i negativna emocio-nalna stanja nezavisni faktori rizika koji utiču na ponovno dobijanje telesne težine nakon LSG (P &lt; 0,05).

**Zaključak:**

Postoji bliska korelacija između gastrointestinal-nih peptidnih hormona i promena težine posle LSG kod pacijenata sa OHS.

## Introduction

Obesity, defined as the abnormal or excessive expansion of white adipose tissue, has reached pandemic proportions and is recognized as a crucial health concern [1]. Extensive clinical research has firmly established that the obese population faces a markedly elevated risk of developing various conditions, including cardiovascular diseases [2], digestive tract disorders [3], infertility [4], and fractures [5], when compared to the general population. At present, the global prevalence of obesity is on an upward trajectory, particularly in developed countries. For example, approximately 42% of the U.S. population has a body mass index (BMI) greater than 30, while 9.4% of the population has a BMI exceeding 4 [6]. While the majority of obese individuals can potentially achieve weight reduction through exercise-based interventions, surgical weight loss procedures are indicated for those with concomitant organ dysfunctions [7]. Among these, obesity hypoventilation syndrome (OHS) stands out as a representative disorder in obese patients. Clinically, it has been well-established that the pathological mechanism of OHS primarily involves a decline in chest wall compliance and restricted diaphragmatic mobility induced by obesity. If not treated promptly, OHS may progress to respiratory failure, thereby posing a serious threat to the patient's life and safety [8]. Given the limitation of respiratory function in OHS patients, exercise-based therapeutic approaches are not suitable for them. With the continuous advancement of medical technology, laparoscopic sleeve gastrectomy (LSG) has witnessed an increasing application in the treatment of OHS in recent years [9]. By modifying the anatomical structure of the gastrointestinal tract and curtailing nutrient intake and absorption, LSG can effectively and rapidly achieve weight reduction and rectify obesity-associated metabolic disorders [10]. Nevertheless, approximately 25% of patients experience weight regain following LSG. Thus, the resolution of this problem remains a pivotal focus of current clinical research.

Recent research indicates that the gastrointestinal tract and the central nervous system interact closely via the autonomic nervous system, the brain-gut peptide transmitter system, and other neuroendocrine networks. This phenomenon is termed the brain-gut axis [11]. Following LSG, significant alterations in the brain-gut axis occur in patients, which may be a crucial factor contributing to weight regain. However, this hypothesis remains unsubstantiated by definitive research. Gastrointestinal peptide hormones are known to play a central role in brain-gut axis interactions by regulating appetite, energy metabolism, gastrointestinal motility, and mood [12]. For example, glucagon-like peptide-1 (GLP-1) suppresses appetite by acting on the arcuate nucleus of the hypothalamus, inhibiting neuropeptide Y (NPY) and acanthamoeba-associated protein (AgRP) neurons, and activating neurons of pheomelanocortino- gen (POMC) [13]. Leptin (Lep) suppresses appetite and increases mood by acting on the hypothalamic leptin receptors, suppressing appetite and increasing energy expenditure [14]. Studies have confirmed that gastrointestinal peptide hormones have a direct relationship with carbohydrate and fat metabolism in humans [15], which suggests the promise of our gastrointestinal peptide hormones as a clinical assessment indicator for regaining weight.

Consequently, this study aims to analyze the factors influencing weight regain after LSG in OHS patients based on gastrointestinal peptide hormones. The findings are expected to offer more reliable and safer clinical guidance for the future implementation of LSG.

## Materials and methods

### Research subjects

This study enrolled patients diagnosed with OHS who underwent LSG at our institution between January 2023 and May 2024. Based on the sample-size calculation formula for sampling surveys, N = (Z^2^ x σ^2^)/d^2^ (Confidence interval Z = 1.96, σ=0.5, overall standard deviation σ=0.5, error d = 10%), a minimum of 96 participants were determined to be necessary for this study. Subsequently, well-defined inclusion and exclusion criteria were formulated.

Inclusion criteria:

- (1) Availability of complete clinical records without missing data.
- (2) BMI > 30 kg/m^2^, with awake arterial carbon dioxide tension (PaCO_2_) ≥ 45 mmHg, accompanied by significant hypoventilation.
- (3) Absence of preoperative interventions that could influence gastrointestinal hormone levels.
- (4) Postoperative percent excess weight loss (EWL%) exceeding 50%.

Exclusion criteria:

- (1) Comorbid conditions such as chronic obstructive pulmonary disease, or pulmonary hypertension that could impair ventilation.
- (2) Active inflammatory or intestinal diseases.
- (3) Diagnosed psychiatric disorders.
- (4) History of gastrointestinal surgery within the preceding six months.
- (5) Use of medications affecting the central nervous system within the past six months.
- (6) Inability to complete follow-up assessments for prognosis evaluation.

After strict screening according to these criteria, a total of 134 subjects were finally enrolled. The study protocol was approved by the Institutional Ethics Committee (Approval number:250303-245), and written informed consent was obtained from all participants. Moreover, the entire research process adheres strictly to the principles outlined in the Declaration of Helsinki.

### Sample collection

Baseline data, including age and gender, were collected from all patients. Body measurements such as BMI, waist circumference, chest circumference, and hip circumference were recorded both preoperatively and on the 7th day postoperatively. Subsequently, 3-5 mL of early-morning elbow venous blood samples were collected from all patients upon admission and 7 days after the surgical procedure. After centrifugal separation of the serum, the concentrations of insulin (INS), Lep, GLP-1, and ghrelin (GHR) were determined using the enzyme-linked immunosorbent assay (ELISA) method.

### Prognostic follow-up

All patients underwent a 6-month prognostic follow-up following LSG. The follow-up was implemented through regular hospital visits at a frequency of once per month. Follow-up included the patient's height and weight, distance from the patient's regained weight. (1) rebound of ≥10%-15% of the lowest postoperative weight; or (2) rebound of more than 25% of the excess preoperative weight; or (3) increase in BMI of ≥5 kg/m^2^ from the lowest postoperative value or BMI≥30 kg/m^2^; or (4) regain of ≥80% of the preoperative weight. Fulfillment of any of the above criteria was judged as regaining weight [16].

### Observation indicators

The alterations in gastrointestinal peptide hormone levels before and after surgery were recorded. Additionally, the predictive mechanism of these gastrointestinal peptide hormones regarding prognostic weight regain was comprehensively analyzed. Finally, factors influencing prognostic weight regain in patients were investigated.

### Statistical analysis

All statistical analyses were performed using SPSS 22.0 software. After the Shapiro-wilk test, it was confirmed that all the measurement data were normally distributed. Measurement data were expressed as mean ± standard deviation (x̄±s), and comparisons between groups were conducted using t-tests or Mann-Whitney U tests. Categorical data were expressed as frequencies and percentages [n(%)], and inter-group comparisons were performed using chi-square tests (χ^2^) or Fisher's exact tests. The Receiver operating characteristic (ROC) curve is used to analyze the predicted value, and the cut-off value is determined based on the largest Youden index scale as well as the Area under curve (AUC). Multivariate logistic regression analysis was employed to identify independent risk factors for weight regain. Statistical significance was set at a *P*-value < 0.05.

## Results

### Changes in gastrointestinal peptide hormones before and after surgery

Compared to preoperative levels, postoperative levels of NS, Lep, and GLP-1 were significantly increased, while GHR levels were decreased (*P* < 0.05, Table 1). These findings indicate a correlation between gastrointestinal peptide hormones and the process of LSG.

**Table 1:** Gastrointestinal peptide hormones before and after treatment. Note: Insulin (INS), leptin (Lep), glucagon-like peptide-1 (GLP-1), and ghrelin (GHR).

	Before treatment (n = 134)	After treatment (n = 134)	t	*P*
INS (pg/mL)	262.18±21.57	317.56±37.33	14.873	<0.001
Lep (pg/mL)	5.84±0.90	7.48±1.36	11.727	<0.001
GLP-1 (pg/mL)	87.12±10.59	100.56±10.36	10.501	<0.001

### Association between gastrointestinal peptide hormones and weight regain

Prognostic follow-up results indicated that 32 out of 134 patients experienced weight regain, corresponding to a weight regain rate of 23.88%. Patients who regained weight demonstrated significantly lower levels of INS, Lep, and GLP-1, as well as elevated levels of GHR, compared to those without weight regain (*P* < 0.05, Table 2). These results suggest that gastrointestinal peptide hormones may be implicated in the mechanisms underlying weight regain.

**Table 2:** Gastrointestinal peptide hormones in patients with and without weight regain.

	Not regaining weight (n = 102)	Weight regains (n=32)	t	*P*
INS (pg/mL)	323.31±36.08	299.25±35.80	3.297	0.001
Lep (pg/mL)	7.70±1.33	6.81±1.24	3.351	0.001
GLP-1 (pg/mL)	102.40±10.30	94.71±8.25	3.847	<0.001
GHR (pg/mL)	223.75±19.42	237.84±17.86	3.647	<0.001

### Predictive value of gastrointestinal peptide hormones for weight regain

**Figure 1:**
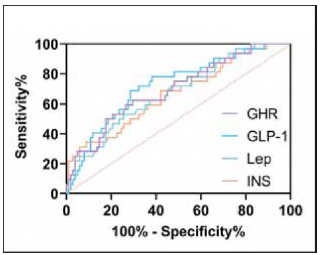
ROC curve of gastrointestinal peptide hormones for predicting weight regain.

ROC curve analysis further revealed that INS, Lep, GLP-1, and GHR all exhibited strong predictive value for post-LSG weight regain (*P* < 0.05, Figure 1 and Table 3). Notably, GLP-1 demonstrated the highest predictive efficacy, with an AUC of 0.724, sensitivity of 68.75%, and specificity of 71.5%.

**Table 3:** Predictive value of gastrointestinal peptide hormones for weight regains. Note: Area Under Curve (AUC).

	Cut-off	Sensitivity (%)	Specificity (%)	AUC	*P*
INS (pg/mL)	<315.60	68.75	57.84	0.665	0.005
Lep (pg/mL)	<6.93	53.13	74.51	0.676	0.003
GLP-1 (pg/mL)	<96.99	68.75	71.57	0.724	<0.001
GHR (pg/mL)	>233.80	62.50	70.59	0.696	<0.001

### Univariate analysis of factors associated with weight regain

Comparative analysis of baseline characteristics between patients with and without weight regain revealed no significant differences in postoperative sex, waist circumference, or chest circumference (*P* > 0.05). However, in the comparison of poor dietary habits, there was a significant difference between re-fatting patients and non-re-fatting patients (*P* < 0.05). At the same time, the number of patients with no exercise habits and micronutrient deficiencies was more than that of patients who had not regained weight, and the SAS and SDS were higher (*P* < 0.05, Table 4).

**Table 4:** Univariate analysis of factors affecting weight regain. Note: Total weight loss (TWL), Systolic Blood Pressure (SBP), Diastolic blood pressure (DBP), Self-Rating Anxiety Scale (SDS), SelfRating Depression Scale (SDS), Abnormal sleep conditions include insomnia, staying up late, and sleeping less than 8h.

	Not regaining weight (n=102)	Weight regains (n=32)	χ^2^/t	*P*
Sex (male/female)	59/43	16/16	0.608	0.436
Ape	48.47±8.14	48.38±5.77	0.062	0.951
TWL (%)	25.03±7.84	26.29±7.96	0.786	0.433
BMI before surgery (kg/m^2^)	35.71±3.00	36.17±2.66	0.785	0.434
Neck circumference (cm)	46.02±4.92	45.94±6.03	0.078	0.938
Chest circumference (cm)	119.67±8.84	122.47±16.42	1.247	0.215
Waist circumference (cm)	119.58±7.95	123.03±12.44	1.852	0.066
Hip circumference (cm)	122.78±12.26	124.78±12.41	0.802	0.424
SBP (mmHp)	138.66±12.38	137.66±17.13	0.362	0.718
DBP (mmHp)	85.75±8.72	87.94±10.78	1.170	0.244
Unhealthy eating habits (often/occasionally/rarely)				
Irregular eating	24/36/42	15/9/8	6.642	0.036
Binge eating	20/39/43	12/15/5	8.5.03	0.014
Nocturnal eating	19/31/52	10/14/8	6.759	0.034
Hiph-sugar/fat foods	24/37/41	12/16/4	8.494	0.014
Smoking (yes/no)	51/51	19/13	0.858	0.354
Sleep (normal/abnormal)	69/33	17/15	2.234	0.135
Exercise habits (yes/no)	39/63	3/29	9.428	0.002
Micronutrients (deficient/normal)	20/82	12/20	4.290	0.038
SAS	31.13±8.94	40.25±8.73	5.064	<0.001
SDS	32.90±8.34	40.00±9.88	4.015	<0.001

### Multivariate analysis of factors associated with weight regain

We assigned values to the above single-factor indicators and gastrointestinal peptide hormones that differed from each other (Table 5), and logistic regression analysis was performed with regaining weight as the independent variable and single-factor indicators as the covariates. The results, as shown in Table 6, showed that gastrointestinal peptide hormones, poor dietary habits, and micronutrient deficiencies were all independent risk factors affecting patients' regaining weight, while maintaining exercise habits was an independent protective factor for regaining weight (*P* < 0.05).

**Table 5:** Assignment of variables for categorization.

Factors	Assignment
Weight regains	No=1, yes=2
Irregular eating, Binge eating, Nocturnal eating, <br>High-sugar/fat foods	Often=3, occasionally=2, rarely=1
Exercise habits	Yes=1, no=2
Micronutrients	Deficient=2, normal = 1
Gastrointestinal peptide hormones, SAS, SDS	Use of raw data

**Table 6:** Multifactorial analysis affecting regaining weight. Note: regression coefficient (B), standard error (S.E), Hazard Ratio [Exp (B)], confidence interval (CI).

Factors	B	S.E	Wals	*P*	Exp (B)	95%Cl
Irregular eating	0.736	1.301	3.543	0.060	1.087	0.002—1.136
Binge eating	3.943	1.468	7.209	0.007	5.566	2.900—16.885
Nocturnal eating	1.940	1.353	2.056	0.152	2.413	0.491—4.583
Hiph-sugar/fat foods	1.975	1.310	12.273	<0.001	2.467	1.533—3.985
Exercise habits	4.216	1.823	5.345	0.021	1.900	1.762—4.790
Micronutrients	1.172	1.151	10.022	<0.001	1.843	1.288—8.034
SAS	0.215	0.077	7.724	0.007	1.240	1.066—1.444
SDS	0.174	0.064	7.403	0.005	1.190	1.050—1.349
INS	-0.024	0.014	13.030	<0.001	0.916	0.642—0.991
Lep	-0.889	0.379	5.502	0.019	0.411	0.196—0.864
GLP-1	-0.108	0.046	5.474	0.019	0.898	0.821—0.983
GHR	0.080	0.43	9.473	<0.001	1.264	1.083—1.843

## Discussion

As obesity becomes increasingly prevalent in modern society, the incidence of OHS has also risen steadily [17]. Although LSG effectively alleviates obesity-related ventilation dysfunction in OHS patients, postoperative weight regain remains a significant concern. To address this issue, this study investigated the factors influencing weight regain in OHS patients, with a focus on gastrointestinal peptide hormones. The key findings are summarized below:

The aim of this study was to assess the likelihood of obesity recurrence in patients at an early stage after receiving LSG by means of gastrointestinal peptide hormone levels, thus providing a clinical reference point and thus early intervention. Therefore, the samples were all collected mainly before and after LSG. Firstly, our observations indicated that following LSG in OHS patients, the levels of INS, Lep, and GLP-1 increased, while GHR levels decreased, suggesting an underlying correlation between these gastrointestinal peptide hormones and obesity progression. Previous research has demonstrated that these hormones modulate appetite and feeding behavior by regulating hypothalamic activity. Specifically, INS, Lep, and GLP suppress appetite and feeding, whereas GHR promotes them. As such, the dysregulation of gastrointestinal peptide hormones is regarded as a key factor contributing to energy metabolism imbalance in obese patients [18]. We posit that LSG, by altering the gastrointestinal anatomy, regulates the levels of these hormones, thereby influencing the gut-brain axis [19]. Additionally, this study revealed a strong correlation between gastrointestinal peptide hormones and post-LSG weight regain, with the hormones showing remarkable predictive value. This can be attributed to the well-established physiological principle that body weight is regulated by hormones [20]. Bariatric-metabolic surgeries induce significant alterations in the normal structure and function of the gastrointestinal tract, as well as modulate the secretion of gut hormones. Research by Carvalho C et al. [21] revealed that patients undergoing LSG and Roux-en-Y gastric bypass exhibit elevated postprandial serum GLP-1 levels, with these changes potentially persisting over the long term. Furthermore, bariatric surgery has been shown to increase levels of peptide YY, suppress the Y2 receptor in the hypothalamic arcuate nucleus, and reduce the expression of gamma-aminobutyric acid, neuropeptide Y, and agouti-related protein. This cascade of effects activates adjacent pro-opiomelanocortin neurons, initiating a series of signaling pathways that suppress appetite and promote weight loss [22]. In addition, altered intestinal flora may affect the production of short-chain fatty acids (SCFAs) [23], and SCFAs may stimulate GLP-1 secretion [24]. If the flora is dysbiot- ic, SCFAs decrease and GLP-1 secretion declines, which may also be one of the mechanisms by which GLP-1 affects weight regain. In essence, gastrointestinal peptide hormones like GLP-1 are crucial for maintaining long-term weight stability in post-surgical patients. Once this balance is disrupted, patients' appetite and nutrient absorption capabilities reactivate, resulting in weight regain [25]. In the study by Çalik Basaran N et al. [26], GLP-1 was identified as a risk factor for weight regain following bariatric surgery, aligning with our findings. Building on these results, Jensen AB et al. [27] have proposed that GLP-1 receptor agonists represent a promising therapeutic strategy for achieving sustained weight control after bariatric-metabolic surgery. Nevertheless, further clinical trials are necessary to validate their efficacy and facilitate broader clinical application.

Apart from gastrointestinal peptide hormones, we must not overlook other factors associated with weight regain. The results of this study indicated that unfavorable dietary habits—including binge eating and a preference for high-sugar and high-fat foods—are risk factors for weight regain. These habits are widely recognized as the most direct lifestyle contributors to obesity [28] [29]. Additionally, a lack of regular exercise is identified as a factor that increases the risk of weight regain. Research by van Baak MA et al. [30] found that patients who engaged in regular physical activity after bariatric-metabolic surgery experienced greater weight loss over time. However, some studies suggest that exercise is not associated with weight regain following such surgeries [31]. This discrepancy may be attributed to the lack of standardized criteria for defining weight regain after bariatric surgery, as well as variations in exercise intensity, duration, and modalities across different studies. Exercise has long been known for its positive effects in improving physical fitness and maintaining health. Regular moderate-intensity exercise improves all aspects of human health and is widely accepted as a preventive and therapeutic strategy for a variety of diseases [32]. Therefore, we prefer to advocate our patients to maintain a good exercise habit. Moreover, trace element deficiencies are also an independent factor influencing weight regain, as trace elements play a critical role in regulating metabolism, hormones, and energy balance in patients. Research has confirmed that trace element deficiencies are prevalent among individuals with obesity [33]. Therefore, addressing these deficiencies is essential for maintaining long-term weight stability and overall health after bariatric-metabolic surgery. Finally, we found that low scores on the Self-Rating Anxiety Scale (SAS) and Self-Rating Depression Scale (SDS) increased the risk of weight regain in patients. Research by Mishali M et al. [34] has shown a link between obesity and depression. Depression is associated with unhealthy lifestyles, uncontrolled eating behaviors, and postbariatric weight regain. Obese patients with comorbid depression and anxiety may exhibit intermittent binge eating behaviors, hampering weight control and worsening psychological issues. In light of these findings, for future LSG procedures, we propose: 1. Monitoring patients' gastrointestinal peptide hormone levels regularly to evaluate postoperative weight regain risk. 2. Enhancing health education for patients, including fostering good dietary and exercise habits. 3. Tracking patients' trace element status and intervening with medications when necessary. 4. Prioritizing patients' psychological well-being, offering guidance and support to alleviate negative emotions.

However, factors like fasting plasma glucose (FPG) and glycated hemoglobin (HbAlc), which have been previously identified in similar studies as factors related to weight regain [35] [36], were not associated with weight regain in this study. This discrepancy may be attributed to the fact that the observation indicators in this study were collected 7 days postoperatively, rather than being real-time data from the patients, which might explain the absence of significant differences. The findings of this paper are more oriented towards offering clinical references for the early assessment of weight regain. Of course, in subsequent research, we intend to incorporate patients' real-time data for validation and further analysis based on the above-mentioned results.

Furthermore, another limitation of this study lies in the relatively small sample size, which may introduce contingency in the analysis results. In the future, it is essential to augment the sample size and prolong the research duration to enhance the reference significance of the research findings. Moreover, incorporating a broader range of observational indicators is necessary to offer more comprehensive assessment insights for clinical weight-regain evaluation. As this study was a retrospective analysis, the prognostic follow-up investigation of the patients was not conducted by members of the research team. The routine follow-up in our hospital only included patients' weight and height, and did not detect gastrointestinal peptides, which also resulted in our inability to add dynamic monitoring of prognostic gastrointestinal peptide hormones. Of course, we cannot ignore the potential impact of medication adherence. Although the postoperative medication was the same for all patients (vitamins + protein supplements + proton pump inhibitor + simethicone), we were unable to monitor the patients' actual daily medication after discharge.

## Conclusion

Gastrointestinal peptide hormones are intricately linked to weight changes in OHS patients after LSG. In the future, the clinic can provide an early assessment of the risk of prognostic weight regain by detecting patients' gastrointestinal peptide hormone levels after LSG, so that early intervention can be managed. Additionally, unfavorable dietary habits, lack of regular exercise, trace element deficiencies, and negative emotional states are identified as independent risk factors influencing weight regain after LSG. Clinicians are thus urged to attach great importance to these factors.

## Dodatak

### Availability of data and materials

The data that support the findings of this study are available from the corresponding author upon reasonable request.

### Funding

This study was supported by the State Key Laboratory of Pathogenesis, Prevention and Treatment of High Incidence Diseases in Central Asia, Xinjiang Medical University (No.SKL-HIDCA-2023-26); Xinjiang Medical University Student Innovation and Entrepreneurship Training Program (No. S202410760073).

### Acknowledgements

Not applicable.

### Conflict of interest statement

All the authors declare that they have no conflict of interest in this work.

## References

[1] Caruso A, Gelsomino L, Panza S, Accattatis F M, Naimo G D, Barone I, et al (2023). Leptin: A Heavyweight Player in Obesity-Related Cancers. Biomolecules.

[2] Iacobellis G (2023). Epicardial fat links obesity to cardiovascular diseases. Prog Cardiovasc Dis.

[3] Aron-Wisnewsky J, Warmbrunn M V, Nieuwdorp M, Clément K (2021). Metabolism and Metabolic Disorders and the Microbiome: The Intestinal Microbiota Associated With Obesity, Lipid Metabolism, and Metabolic Health-Pathophysiology and Therapeutic Strategies. Gastroenterology.

[4] Marinelli S, Napoletano G, Straccamore M, Basile G (2022). Female obesity and infertility: outcomes and regulatory guidance. Acta Biomed.

[5] Kupisz-Urbańska M, Stuss M, Kuryłowicz A, Jankowski P, Pilz S, Sewerynek E, et al (2022). Fracture risk in obesity: a narrative review. Endokrynol Pol.

[6] Kacmarek R M, Wanderley H V, Villar J, Berra L (2021). Weaning patients with obesity from ventilatory support. Curr Opin Crit Care.

[7] Ma Q, He X, Fu Z, Ren X, Sun R, Zhu S, Bian Y, Li X (2024). Clinical observation of laparoscopic sleeve gastrectomy and metformin treatment in obese PCOS patients. J Med Biochem.

[8] Orozco Gonzalez B N, Rodriguez Plascencia N, Palma Zapata J A, Llamas Dominguez A E, Rodriguez Gonzalez J S, Diaz J M, et al (2024). Obesity hypoventilation syndrome, literature review. Sleep Adv.

[9] Climaco K, Ahnfeldt E (2021). Laparoscopic Vertical Sleeve Gastrectomy. Surg Clin North Am.

[10] Clarysse M, Van Aelst P, Vanuytsel T, Monbaliu D, Ceulemans L J, Mertens A, et al (2023). Laparoscopic Sleeve Gastrectomy for Obesity After Combined Liver-intestinal Transplantation: A Case Report. Transplantation.

[11] Mayer E A, Nance K, Chen S (2022). The Gut-Brain Axis. Annu Rev Med.

[12] Albrechtsen N J W, Rehfeld J F (2021). On premises and principles for measurement of gastrointestinal peptide hormones. Peptides.

[13] Newsome P N, Ambery P (2023). Incretins (GLP-1 receptor agonists and dual/triple agonists) and the liver. J Hepatol.

[14] Obradovic M, Sudar-Milovanovic E, Soskic S, Essack M, Arya S, Stewart A J, et al (2021). Leptin and Obesity: Role and Clinical Implication. Front Endocrinol (Lausanne).

[15] Lundanes J, Storliløkken G E, Solem M S, Dankel S N, Tangvik R J, Ødegård R, et al (2025). Gastrointestinal hormones and subjective ratings of appetite after low-carbohydrate vs low-fat low-energy diets in females with lipedema - A randomized controlled trial. Clin Nutr ESPEN.

[16] Noria S F, Shelby R D, Atkins K D, Nguyen N T, Gadde K M (2023). Weight Regain After Bariatric Surgery: Scope of the Problem, Causes, Prevention, and Treatment. Curr Diab Rep.

[17] Paranicova I, Bodnarova S, Trojova I, Hertelyova Z, Gulasova Z, Cimbolakova I, et al (2024). Long-term myocardial effects of noninvasive ventilation in patients with obesity hypoventilation syndrome. Respir Med.

[18] Santos-Hernandez M, Reimann F, Gribble F M (2024). Cellular mechanisms of incretin hormone secretion. J Mol Endocrinol.

[19] Iriondo-DeHond A, Uranga J A, Del Castillo M D, Abalo R (2020). Effects of Coffee and Its Components on the Gastrointestinal Tract and the Brain-Gut Axis. Nutrients.

[20] Farhadipour M, Depoortere I (2021). The Function of Gastrointestinal Hormones in Obesity-Implications for the Regulation of Energy Intake. Nutrients.

[21] Carvalho C, de Souza A L, Batista G A, Duran L F T, Fernandes D P, Molina V B C, et al (2022). GLP-1: 10-year follow-up after Roux-en-Y gastric bypass. Langenbecks Arch Surg.

[22] Patkar P P, Hao Z, Mumphrey M B, Townsend R L, Berthoud H R, Shin A C (2019). Unlike calorie restriction, Roux-en-Y gastric bypass surgery does not increase hypothalamic AgRP and NPY in mice on a high-fat diet. Int J Obes (Lond).

[23] Wang J, Zhu N, Su X, Gao Y, Yang R (2023). Gut-Microbiota-Derived Metabolites Maintain Gut and Systemic Immune Homeostasis. Cells.

[24] Zhang D, Jian Y R, Zhang Y N, Li Y, Gu L T, Sun H H, et al (2023). Short-chain fatty acids in diseases. Cell Commun Signal.

[25] Kellett J, Soliman S S, Podwojniak A, Minkanic M, Kumar G, Goodwin B, et al (2025). The Efficacy of Glucagon-like Peptide-1 (GLP-1) Receptor Agonists for Insufficient Weight Loss or Regain After Metabolic/Bariatric Surgery: A Systematic Review and Meta-analysis. Obes Surg.

[26] Calik Basaran N, Dotan I, Dicker D (2024). Post metabolic bariatric surgery weight regain: the importance of GLP-1 levels. Int J Obes (Lond).

[27] Jensen A B, Renström F, Aczél S, Folie P, Biraima-Steinemann M, Beuschlein F, et al (2023). Efficacy of the Glucagon-Like Peptide-1 Receptor Agonists Liraglutide and Semaglutide for the Treatment of Weight Regain After Bariatric surgery: a Retrospective Observational Study. Obes Surg.

[28] van Baak M A, Mariman E C M (2023). Obesity-induced and weight-loss-induced physiological factors affecting weight regain. Nat Rev Endocrinol.

[29] Cifuentes L, Galbiati F, Mahmud H, Rometo D (2024). Weight regain after total meal replacement very low-calorie diet program with and with-out anti-obesity medications. Obes Sci Pract.

[30] van Baak M A, Pramono A, Battista F, Beaulieu K, Blundell J E, Busetto L, et al (2021). Effect of different types of regular exercise on physical fitness in adults with overweight or obesity: Systematic review and meta-analyses. Obes Rev.

[31] Oppert J M, Bellicha A, Ciangura C (2021). Physical activity in management of persons with obesity. Eur J Intern Med.

[32] Qiu Y, Fernández-García B, Lehmann H I, Li G, Kroemer G, López-Otín C, et al (2023). Exercise sustains the hallmarks of health. J Sport Health Sci.

[33] Thibault R, Pichard C (2016). Overview on nutritional issues in bariatric surgery. Curr Opin Clin Nutr Metab Care.

[34] Mishali M, Kisner M (2022). Psycho-behavioral Factors Related to Weight Regain After Bariatric Surgery. Obes Surg.

[35] Çeler Ö, Er H C, Sancak S, Çırak E, Özdemir A, Sertbaş Y, et al (2023). The Effects of Laparoscopic Sleeve Gastrectomy (LSG) on Obesity-Related Type 2 Diabetes Mellitus: a Prospective Observational Study from a Single Center. Obes Surg.

[36] Varma S, Clark J M, Schweitzer M, Magnuson T, Brown T T, Lee C J (2017). Weight regain in patients with symptoms of post-bariatric surgery hypoglycemia. Surg Obes Relat Dis.

